# The Role of the Microenvironment and Cell Adhesion Molecules in Chronic Lymphocytic Leukemia

**DOI:** 10.3390/cancers15215160

**Published:** 2023-10-26

**Authors:** Marina Cerreto, Robin Foà, Alessandro Natoni

**Affiliations:** Hematology, Department of Translational and Precision Medicine, Sapienza University, 00100 Rome, Italy; marina.cerreto@uniroma1.it (M.C.); rfoa@bce.uniroma1.it (R.F.)

**Keywords:** CLL, cell adhesion molecules, transendothelial migration, CAMDR, microenvironment, selectins, integrins, CD49d, Bruton tyrosine kinase inhibitors, targeted therapy

## Abstract

**Simple Summary:**

Progression of chronic lymphocytic leukemia (CLL) and its response to therapies are largely dependent on the microenvironment of the bone marrow and lymph nodes, which nurtures leukemic cells and protects them from therapeutic agents. Hence, cell trafficking between the blood vessels and lymphatic tissues is critical for CLL pathophysiology. Cell adhesion molecules mediate the re-localization of CLL cells in different anatomical compartments and are involved in their survival and proliferation. Evaluation of the molecular mechanisms underlying their activation and functions has uncovered clinically relevant signaling pathways targeted by well-established and new therapeutic strategies. The aim of this review is to summarize the current knowledge regarding the microenvironment and the cell adhesion molecules that have been shown to be important in CLL and their role in transendothelial migration and cell adhesion-mediated drug resistance. We also discuss how novel therapeutic compounds modulate the functions of this important class of molecules.

**Abstract:**

Chronic lymphocytic leukemia (CLL) is a B-cell malignancy whose progression largely depends on the lymph node and bone marrow microenvironment. Indeed, CLL cells actively proliferate in specific regions of these anatomical compartments, known as proliferation centers, while being quiescent in the blood stream. Hence, CLL cell adhesion and migration into these protective niches are critical for CLL pathophysiology. CLL cells are lodged in their microenvironment through a series of molecular interactions that are mediated by cellular adhesion molecules and their counter receptors. The importance of these adhesion molecules in the clinic is demonstrated by the correlation between the expression levels of some of them, in particular CD49d, and the prognostic likelihood. Furthermore, novel therapeutic agents, such as ibrutinib, impair the functions of these adhesion molecules, leading to an egress of CLL cells from the lymph nodes and bone marrow into the circulation together with an inhibition of homing into these survival niches, thereby preventing disease progression. Several adhesion molecules have been shown to participate in CLL adhesion and migration. Their importance also stems from the observation that they are involved in promoting, directly or indirectly, survival signals that sustain CLL proliferation and limit the efficacy of standard and novel chemotherapeutic drugs, a process known as cell adhesion-mediated drug resistance. In this respect, many studies have elucidated the molecular mechanisms underlying cell adhesion-mediated drug resistance, which have highlighted different signaling pathways that may represent potential therapeutic targets. Here, we review the role of the microenvironment and the adhesion molecules that have been shown to be important in CLL and their impact on transendothelial migration and cell-mediated drug resistance. We also discuss how novel therapeutic compounds modulate the function of this important class of molecules.

## 1. Introduction: The Importance of the Microenvironment in CLL

Chronic lymphocytic leukemia (CLL) is a B-cell malignancy characterized by the accumulation of CD5^+^ B cells in the peripheral blood (PB), bone marrow (BM), and lymph nodes (LNs). Typically, CLL cells are small, mature-appearing lymphocytes with a dense nucleus and a thin cytoplasm, expressing CD5, CD19, CD23, CD200, and low levels of CD20. Clinically, CLL can be divided into two main subsets based on the presence of mutations in the immunoglobulin heavy-chain variable region gene (*IGHV*) [1]. CLL cells with mutated *IGHV*s originate from a B cell that has undergone differentiation in the germinal centers of the LN, where B cells undergo somatic hypermutation in their *IGHV* genes and selection during an immune response. CLL cells expressing unmutated *IGHV*s arise from a B cell that has not undergone differentiation. CLL patients with unmutated *IGHV*s typically experience a more aggressive disease than patients with mutated *IGHV*s [2].

Although for many years CLL was referred to as a disease of “failed apoptosis”, pioneering experiments measuring deuterium (^2^H) incorporation in vivo in CLL patients have shown that a fraction of CLL is enriched in actively dividing cells [3], which are mainly found in specific anatomical compartments of the LNs known as “proliferation centers” or “pseudofollicles” [4,5]. Indeed, CLL cells circulating in the PB are quiescent and arrested in the G0/G1 phase of the cell cycle, whereas those residing in the proliferation centers express markers of cell proliferation and DNA replication such as Ki67 [6], cyclin D1 [7,8], and cyclin D2 [9], and phosphorylation of the minichromosome maintenance 2 (pMCM2) [10]. Although malignant B cells tend to accumulate in the PB in vivo, CLL cells undergo spontaneous and rapid apoptosis when cultured in vitro, clearly demonstrating that in order to survive, they require proper humoral and cellular factors provided by the microenvironment [11].

The tumor microenvironment consists of different components such as stromal cells, immune cells, extracellular matrix proteins, and soluble factors that promote cancer cell survival, growth, and resistance to cell death [12]. Pro-survival signals are transmitted by secreted molecules or by cell–cell interactions. In CLL, malignant cells are sustained by a variety of soluble and membrane-bound factors that emanate from the surrounding environment, which include B-cell receptor (BCR) engagement by foreign and self-antigens [13], activation of the G protein-coupled C-X-C motif chemokine receptor 4 (CXCR4) by the stromal cell-derived factor-1α (SDF-1α), also known as C-X-C motif chemokine ligand (CXCL) 12 [14], stimulation of the tumor necrosis factor (TNF) receptors by their cognate ligands, such as CD40 [15,16,17,18] and B-cell activating factor (BAFF) receptors [19,20], and signals elicited by interleukin (IL)-4 [21,22,23,24], IL-15 [25,26,27] and IL-21 [28,29]. All these signals are responsible for CLL proliferation as well as chemotherapy resistance, as they trigger anti-apoptotic pathways that protect CLL cells from standard [30,31] and novel anti-leukemic agents, including venetoclax [32,33]. Moreover, the microenvironment and cell-to-cell crosstalk may also contribute to an increase in the CLL autocrine production of cytokines and ILs, such as IL-2 [34], IL-8 [35,36], and TNF-α [37,38], that have been shown to be involved in CLL survival and disease progression.

Not only are CLL cells nurtured by the microenvironment, but they also actively shape it to their own advantage, establishing the optimal conditions for disease progression [39]. One of the most striking features of CLL is the presence of a population of pro-tumor, M2-like macrophages termed nurse-like cells (NLCs), which support CLL cell survival by inhibiting spontaneous as well as drug-induced apoptosis [40]. It is now clear that CLL cells are the primary drivers of skewing CD14^+^ monocytes towards an NLC phenotype, as CD14^+^ monocytes fail to differentiate into NLCs when co-cultured with normal B cells [41,42]. CLL cells actively contribute to establishing an immuno-tolerant microenvironment by secreting IL-6 and IL-10, which in turn suppress the T-cell response [43,44,45]. It has been shown that the exosomes and extracellular vesicles produced by CLL cells also participate in shaping the microenvironment towards pro-inflammatory and immuno-suppressive phenotypes [46,47] by delivering microRNAs and proteins such as mir-146a and mir-451 [48], mir-150 [49], and the anexelekto (Axl) receptor tyrosine kinase [50]. In CLL, the microenvironment, and in particular, the presence of specific immune cells, has a significant prognostic value. For instance, high levels of regulatory T cells (Treg) have been associated with a poorer prognosis and shorter survival [51]. Similarly, an increased number of the circulating γδ T-cell subset Vγ9Vδ2 correlates with a shorter time-to-first treatment, especially in patients with unmutated *IGHV*s [52].

Given the dependence of CLL cells on the microenvironment, much effort has been devoted to the rational design of therapeutic compounds targeting the interactions between CLL cells and their microenvironment. This has led to the development of clinically relevant drugs such as ibrutinib, a Bruton’s tyrosine kinase (BTK) inhibitor, which causes the release of malignant cells from the protective LN and BM niches into the PB, where they are deprived of proliferative signals and become more susceptible to cell death [53].

## 2. Components of the CLL Microenvironment

**Figure 1:**
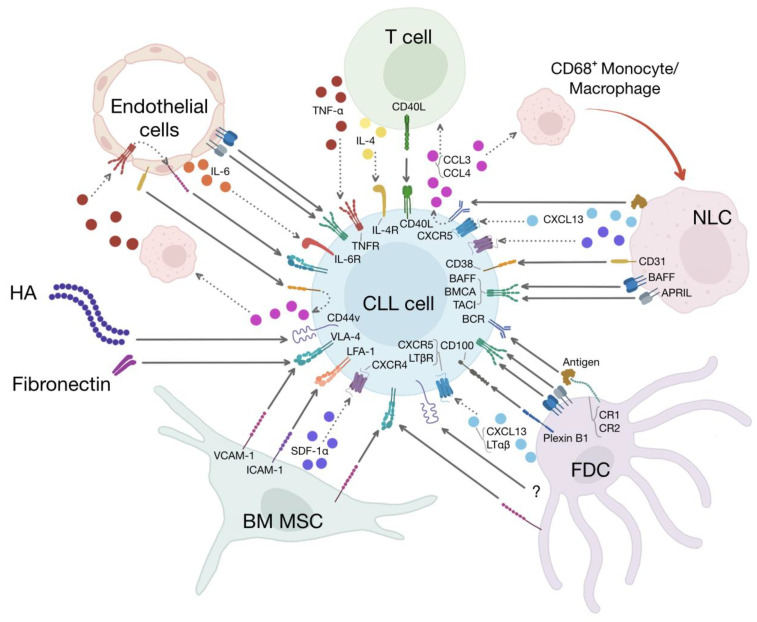
The CLL microenvironment. Different types of cells and proteins of the extracellular matrix constitute the CLL microenvironment. The cellular components of this microenvironment include BM MSCs, FDCs, NLCs, T cells, and endothelial cells. The extracellular matrix is composed of, among others, fibrillar proteins such as fibronectin and hyaluronic acid (HA). All these different elements of the CLL microenvironment establish unidirectional as well as bidirectional interactions with CLL cells, leading to CLL migration, immune suppression, and recruitment of accessory cells that participate in CLL survival and proliferation. Moreover, activation of the adhesion molecules expressed on CLL cells induces chemoresistance through a process known as cell-mediated drug resistance. BM MSCs express VCAM-1 and ICAM-1, which stimulate CLL adhesion and survival through the binding to VLA-4 and LFA-1, respectively. BM-MSCs also secrete SDF-1α, leading to CLL migration and survival. FDCs contribute to CLL survival through a diverse array of membrane-bound and soluble molecules that include VCAM-1/VLA-4 interactions, ligation of CD44, secretion of CXCL13 and LTαβ, stimulation of the BCR by unprocessed antigens presented by CR1 and CR2, plexin B1/CD100 interactions and pro-survival factors of the TNF family such as BAFF and APRIL, which activate their cognate receptors (BAFFR, BCMA, and TACI) present on the surface of CLL cells. NLCs are specialized tumor-supporting, M2-like, CD68^+^ macrophages that induce CLL migration and survival by secreting SDF-1α, CXCL13 and presenting to CLL cells antigens and pro-survival molecules such as BAFF, APRIL, BDNF (not shown), and CD31, which is the ligand for CD38 expressed on CLL cells. Stimulation of BCR by NCLs induces the release of CCL3 and CCL4 by CLL cells, which in turn recruit supportive T cells and further CD68^+^ monocytes/macrophages in the microenvironment. T cells induce CLL proliferation and survival through CD40/CD40L interactions and IL-4 and TNF-α secretion. Endothelial cells are part of a vicious cycle established by the interactions between endothelial CD31 and CD38, which induce the release of CCL3 and CCL4 by the malignant cells. As previously described, these chemokines recruit CD68^+^ monocytes/macrophages that activate the endothelium through the secretion of TNF-α. Activated endothelial cells express VCAM-1, which induces CLL survival through VLA-4 engagement. Other pro-survival factors provided by endothelial cells include IL-6, BAFF, and APRIL. HA and fibronectin stimulate CLL survival through CD44 and VLA-4, respectively.

In the lymphoid and marrow niches, CLL cells establish contact with the fibrillary components of the extracellular matrix and many types of specialized cells originating from the hematopoietic as well as mesenchymal lineages. Among these are the BM mesenchymal stromal cells (BM MSCs), the follicular dendritic cells (FDCs), the NLCs, T cells, and endothelial cells (ECs) (Figure 1). In this section, we discuss these different components of the CLL microenvironment and how they contribute to CLL survival and resistance to therapy.

### 2.1. Bone Marrow Mesenchymal Stromal Cells

BM MSCs are a rare population of multipotent progenitor cells that modulate B-cell proliferation and differentiation and contribute to the normal BM architecture. In CLL, they are present in secondary lymphatic tissues and represent the main source of SDF-1α, a chemokine with pleiotropic effects in CLL cells [13,19]. Indeed, SDF-1α coordinates CLL transendothelial migration and tissue homing by regulating the function of molecules involved in cell motility and adhesion [14,54], such as integrins [55], and also promotes CLL survival [19,40]. Moreover, in vitro, the SDF-1α produced by BM MSCs induces a spontaneous migration of CLL cells beneath the BM MSCs layer, a process known as pseudoemperipolesis [54]. BM MSCs also secrete high levels of IL-1α, IL-1β, IL-15, and transforming growth factor β-1 (TGFβ-1), thereby rescuing CLL cells from apoptosis [19,56]. Moreover, BM MSCs protect CLL cells from spontaneous and drug-induced apoptosis through direct cell–cell contacts, a process also known as cell adhesion-mediated drug resistance (CAMDR) [31,57]. Adhesion of CLL cells to BM MSCs is primarily mediated by β1 and, to a lesser extent, β2 integrins [57,58]. BM MSCs express the vascular cell adhesion molecule-1 (VCAM-1), which represents one of the most important receptors for the very late antigen-4 (VLA-4), also known as α4β1 integrin. The interactions between VCAM-1 present on BM MSCs and VLA-4 expressed on CLL cells mediate the retention of leukemic cells in the BM microenvironment, thus promoting drug resistance [55,58]. BM MSCs can also protect CLL from spontaneous and drug-induced apoptosis by increasing the ability of leukemic cells to maintain the intracellular redox balance [59]. Indeed, CLL cells display high levels of reactive oxygen species (ROS); therefore, they are particularly dependent on cellular antioxidants, such as glutathione (GSH), to counteract the cytotoxic effects of endogenous ROS [60]. BM MSCs promote the GSH metabolism by releasing cysteine into the microenvironment, which in turn is used by CLL cells to synthesize GSH [59]. Another mechanism by which BM MSCs promote CLL survival is the induction of a glycolytic shift in CLL cells through the neurogenic locus notch homolog protein 1 (Notch)/c-Myc signaling pathways. Finally, it has been shown that BM MSCs induce the expression of markers that correlate with aggressive disease, such as zeta-chain-associated protein kinase-70 (ZAP-70) and CD38 [61] together with a downregulation of CD20, which may be implicated in resistance to anti-CD20 antibody treatment [62]. All these studies indicate that the BM MSCs have multiple mechanisms capable of sustaining CLL survival, including CAMDR and metabolic reprogramming.

### 2.2. Follicular Dendritic Cells

FDCs are specialized cells of stromal origin primarily resident in the germinal centers of lymphoid tissues [63]. Here, they interact with other stromal cells, such as fibroblasts and ECs, creating a complex network that supports lymphocyte survival, migration, and activation [64]. In the *IGH* enhancer T-cell leukemia-1 oncogene (*Eμ-Tcl1*) transgenic mouse model of CLL, it has been shown that the crosstalk between FDCs and tumor B cells is essential to supporting the consecutive steps of leukemia pathogenesis by inducing a proper localization of CLL-like tumor cells within lymphoid follicles [65]. This crosstalk is mediated by the CXCL13–CXCR5 and the lymphotoxin beta receptor (LTβR)/lymphotoxin alpha beta (LTαβ) signaling axes. FDCs inhibit spontaneous apoptosis in vitro through direct cell–cell interactions that are mediated by the ligation of CD44 on CLL cells [66] and the engagement of semaphoring CD100 expressed on CLL cells and its counter receptor plexin B1 [67]. Moreover, FDCs are capable of sustaining CLL proliferation and survival by many other mechanisms, including BCR activation on CLL cells during the presentation of unprocessed antigens via complement receptors 1 (CR1/CD35) and 2 (CR2/CD21) and the expression of survival factors such as BAFF and a proliferation-inducing ligand (APRIL) and molecules involved in CAMDR, including VCAM-1 [39]. These studies highlight that FDCs are an important component of the stromal microenvironment, particularly in secondary lymphoid organs.

### 2.3. Nurse-like Cells

NLCs are specialized cells found in the lymphoid tissues of CLL patients that derive from monocytes and share similarities with macrophages [40]. NLCs express variable levels of CD14, CD33, CD45, and the human leukocyte antigen-DR isotype (HLA-DR) and are negative for CD3, CD40L, and VCAM-1 [68]. These cells have been considered as CLL-specific tumor-associated macrophages with an M2-like phenotype, characterized by high levels of CD68 and CD163 expression [69] and impaired phagocytic activity [70]. Indeed, NCLs have immunosuppressive properties as they inhibit T-cell proliferation while driving the expansion of Treg [71]. It has been shown that CLL cells promote NLC differentiation in the tumor microenvironment by releasing soluble factors such as nicotinamide phosphoribosyl transferase (NAMPT) and the nuclear protein high-mobility group protein B1 (HMGB1) [41,42]. NLCs secrete in the tumor microenvironment different pro-survival factors for CLL cells, including SDF-1α [40], CXCL13 [72], BAFF, and APRIL [73]. They also express antigens, which can activate the BCR [74], and CD31, the ligand for CD38 present on CLL cells, which cooperates with plexin-B1, also expressed on NLCs, in delivering pro-survival signals [75]. It has been shown that stimulation of the BCR by NLC-derived antigens induces the secretion of C-C motif chemokine ligand (CCL) 3 and CCL4, chemokines that promote the recruitment of additional CD68^+^ monocyte/macrophages and, to a lesser extent, T cells [76]. Interestingly, CCL3 and CCL4 are mainly produced by CLL cells carrying bad prognosticator, such as CD38 and CD49d [77]. An additional pro-survival pathway has been recently described whereby NLCs secrete brain-derived neurotrophic factor (BDNF), leading to the activation of the neurotensin receptor 2/tropomyosin-related kinase receptor B (NTSR2/TrkB) complex on CLL cells and the subsequent induction of anti-apoptotic proteins [78,79]. NCLs contribute to CLL retention in the LNs through the expression of CCL21 [80]. This chemokine is present on the surface of NCLs and interacts with CCR7 expressed by CLL cells, promoting the retention of malignant cells within the LNs. Interestingly, it has been shown that CCL21/CCR7 interactions induce VLA-4 and lymphocyte function-associated antigen-1 (LFA-1) inside-out activation and clustering, which further contribute to CLL lodging in the LNs [81].

### 2.4. T Cells

T cells have a significant impact on the development, progression, and immune response against CLL cells. Overall, the number of T cells is rather high in untreated CLL patients, with a prevalence of CD8^+^ T cells in the PB and CD4^+^ T cells in the LN and BM [82]. Despite this, T cells appear to be non-functional [82], display reduced proliferative and cytotoxic potential [83], have impaired capacity to induce differentiation of B-lymphocytes [84], and express markers of exhaustion, such as cytotoxic T-lymphocyte antigen-4 (CTLA-4) [85] and programmed cell death protein 1 (PD-1) [83]. Interestingly, CLL cells prevent fully immuno-competent CD8^+^ T cells from establishing a proper polarization of their lytic granules (non-lytic degranulation), leading to an abnormal immune synapse, a feature that is also shared by normal B cells [86]. Notably, CLL cells are capable of inducing non-lytic degranulation also in healthy allogeneic T cells [87]. CLL cells drive these T-cell abnormal phenotypes by releasing factors such as IL-10 and TGF-β1, which impair T-cell activation and effector functions, ultimately facilitating CLL cell survival and proliferation [88]. Moreover, CLL cells induce abnormal maturation and functional impairment of circulating dendritic cells in part through the secretion of IL-6, which contributes to a defective T-cell response [89].

Although functionally impaired, T cells retain the ability to release cytokines, such as TNF-α, that induce CLL proliferation and inhibit apoptosis [83]. Within the proliferation centers, a considerable proportion of T cells, mostly belonging to the CD4^+^ subset, are in close proximity to proliferating Ki67^+^ CLL cells [90]. These CD4^+^ T cells express the CD40 ligand (CD40L) and migrate into the proliferation centers in response to CCL17 and CCL22 secreted by proliferating CLL cells [91]. Stimulation of CLL cells by CD40L and IL-4, which is also secreted by CD4^+^ T cells, induces proliferation, rescues leukemic cells from spontaneous apoptosis, and induces chemoresistance [10,16,18,29,30,33,92]. Therefore, it seems that CD4^+^ CD40L^+^ T cells take part in a vicious cycle within the pseudofollicles whereby they promote CLL proliferation, which in turn releases cytokines to attract additional CD4^+^ CD40L^+^ T cells. The importance of the CD4^+^ T cell population is also demonstrated by the evidence that in vivo is required for proper engraftment of leukemic B cells in patient-derived xenograft models [93].

### 2.5. Endothelial Cells

ECs are an integral component of the LN and BM microenvironments and establish intimate connections with CLL cells, in particular with those cells expressing molecular markers of proliferation and differentiation, including Ki67 and activation-induced cytidine deaminase (AID), respectively [94,95]. CLL cells stimulate endothelial proliferation and angiogenesis by releasing pro-angiogenic factors such as vascular endothelial growth factor (VEGF) and angiopoietin 2 (Ang2) [95,96,97]. Moreover, LN and BM from CLL patients display abnormal vascular elements that are related to disease stage and are predictors of poor clinical outcomes [98,99,100,101]. ECs release different soluble as well as membrane-bound factors, including BAFF and APRIL [94], IL-6 dimers [102], and VCAM-1 [103], that promote the survival of CLL cells. Indeed, culturing CLL cells with ECs in vitro increases the levels of anti-apoptotic proteins, such as B-cell lymphoma-2 (Bcl-2), Bcl-extra-large (Bcl-xL), and myeloid leukemia cell differentiation protein-1 (Mcl-1), as well as CD38 and CD49d, in a nuclear factor-κB (NF-κB)-dependent manner [104]. Interestingly, the interaction between CLL and ECs establishes different types of reciprocal cross-talk, leading to the consolidation of vicious cycles that ultimately promote CLL survival and resistance to therapy. For instance, the interaction between CD40L expressed by CLL cells and CD40 expressed on ECs stimulates the secretion of endothelial-derived BAFF and APRIL, which in turn induce CLL activation, proliferation, and survival [94]. Moreover, CD31 present on ECs engages CD38 on CLL cells, inducing the release of CLL-derived CCL3 and CCL4 [77]. These chemokines recruit CD68^+^ monocytes/macrophages that release TNF-α, leading to the expression of VCAM-1 on ECs, which sustains CLL survival [77]. Moreover, engagement of the endothelin subtype A receptor (EtAR) expressed on CLL cells by endothelin-1 (ET-1), secreted by ECs, induces proliferation and chemo-resistance in CLL, indicating the presence of an additional survival pathway [105]. All these data demonstrate that ECs are not only a passive barrier that CLL cells must cross to migrate into protective niches but also play an active role in delivering pro-survival and proliferative signals.

### 2.6. Extracellular Matrix

The non-cellular component of secondary lymphoid tissues, the extracellular matrix (ECM), plays a critical role in promoting CLL survival in the microenvironment. Not only does the ECM provide the scaffold that allows CLL cells to interact with the cellular component of the microenvironment, but it also represents a reservoir of growth factors and chemokines [106,107]. The ECM consists of glycoproteins such as fibronectin and laminin that are ligands for the α4β1 and α3β1 integrins expressed by CLL cells [108,109]. Engagement of these integrins by the ECM induces apoptosis resistance and promotes the dissemination of CLL lymphocytes through vascular basement membranes and possibly LN compartments.

## 3. Transendothelial Migration

Migration of leukocytes from the bloodstream into the lymphoid tissue, commonly known as transendothelial migration (TEM), is a multistep process whereby cells of the immune system first slow their flow in order to tether and roll on the vessel walls of ECs, priming them for chemokine recognition, which in turn leads to firm adhesion and subsequent extravasation [110]. Different types of molecules take part in this process, such as soluble factors released by the microenvironment and adhesion molecules expressed by endothelial/stromal cells or interspersed in the ECM. Adhesion molecules include membrane-bound glycoproteins and glycosylated fibrillary proteins.

The first step of the TEM is mediated by a class of molecules called selectins and their natural ligands expressed on the luminal surface of the endothelium. Selectins constitute a family of calcium-dependent (C-type) lectins that recognize and bind carbohydrate determinants, decorating proteins and lipids present on the cell surface [111]. Three types of selectins have been identified so far. P-selectin (CD62P) is stored in both the α-granules of platelets and in the Weibel–Palade bodies of ECs. These vesicles promptly translocate P-selectin on the cell surface following stimulation by different environmental cues, allowing a rapid response to cellular damage. E-selectin (CD62E) is not pre-formed, and its expression is transcriptionally induced by pro-inflammatory cytokines such as TNF-α, LPS, and IL-1. E-selectin is also constitutively expressed in the BM microvessels, where it is critical for the recruitment and lodging of hematopoietic stem cells (HSCs) [112]. Finally, L-selectin (CD62L) expression is restricted to leukocytes and HSCs. Importantly, L-selectin requires a hydrodynamic shear threshold to establish proficient binding with its counter receptors, below which no interactions are observed [113]. Selectins mediate the slow tethering and rolling of leukocytes on the endothelium, a process that requires the rapid association and dissociation of selectin–ligand bonds [114]. This process is carbohydrate dependent, as shown by leukocyte adhesion deficiency II (LAD II), a rare disorder whereby a mutation in the gene encoding for the guanosine 5′-diphosphate (GDP)-fucose transporter, localized in the Golgi apparatus, leads to a complete inhibition of selectin ligand synthesis and the subsequent disruption of selectin-mediated leukocyte adhesion [115]. The minimal carbohydrate structure required for selectin binding is a tetrasaccharide molecule containing sialic acid and fucose that is sialyl-Lewis x (sLe^x^) and its isomer sialyl-Lewis a (sLe^a^). The carbohydrate determinants recognized by the endothelial E- and P-selectins are typically exposed to their leukocyte counter receptors, which are specific glycoforms of proteins and lipids. Among the protein carriers there is the cutaneous lymphocyte antigen (CLA), which is a glycoform of P-selectin glycoprotein ligand-1 (PSGL-1/CD162), the hematopoietic cell E-/L-selectin ligand (HCELL), which is a glycoform of CD44, the glycoform of CD43, and L-selectin, which can function as an E-selectin ligand [116]. Specificity towards a particular selectin is determined by the presence of additional modifications such as sulfation.

The slow rolling mediated by selectins allows leukocytes to bind local chemokines, leading to the activation of integrins, a different class of molecules responsible for firm adhesion. Once activated, integrins can recognize and bind their ligands, determining the arrest of the leukocytes on endothelial cells. Owing to this property, integrins are defined as “activatable” receptors since intracellular signaling is required to greatly increase their capability to bind their ligands [117]. Integrins are a superfamily of highly glycosylated, allosterically regulated, heterodimeric transmembrane receptors that mediate cell–cell and cell–matrix adhesion. In mammals, 24 distinct combinations of α- and β-subunits have been described [118]. In humans, there are 18 different isoforms for the α subunit and 8 for the β subunit.

Integrin ligands are mostly present in the extracellular matrix and on the cell surface, although some can also be soluble. Integrins are not constitutively capable of binding their ligands; their activity is regulated by conformational changes induced from inside the cell, through a process called inside-out signaling. Large-scale rearrangements shift between three different conformations: Two forms with a low affinity for ligands (a bent form and an extended form) and one with a higher affinity [119]. Integrins are maintained in a bent, low-affinity state through weak interactions between the transmembrane and cytoplasmic domains of the α and β subunits. Signals disrupting these interactions cause the legs of the α and β ectodomains to move apart so that they can unbend [120]. This mechanism leads to affinity upregulation, extension, and clustering that result in an increase in cellular adhesiveness. Most of the signals that activate integrins come from chemokines that bind to their G protein-coupled receptors (GPCRs) and activate Rho-family GTPases and effectors [119]. After this activation process, strong integrin-mediated interactions allow leukocytes to migrate through the endothelium to the underlying tissues.

## 4. Cell Adhesion Molecules in CLL

**Figure 2:**
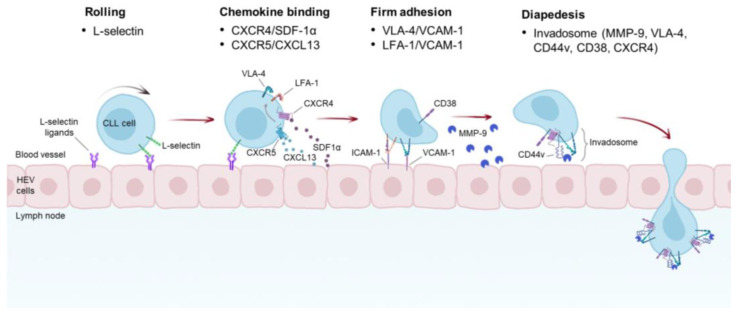
Proposed mechanism of CLL TEM into the LNs. CLL cells roll onto the walls of the high endothelial venules (HEVs) of the LNs. This rolling is mediated by L-selectin expressed on CLL cells and its ligands present on the luminal surface of HEVs. The tethering and rolling of CLL cells enable them to bind chemokines secreted by HEV and underlying cells, including NLC, BM MSC, and FDCs. SDF-1α and CXCL13 have been shown to be the main chemokines promoting VLA-4 and LFA-1 activation by an inside-out signaling. Moreover, autocrine production of VEGF by CLL cells promotes SDF-1α- and CCL21-mediated TEM through the activation of VLA-4 (not shown). Triggering of VLA-4 and LFA-1 induces binding of these integrins to their cognate receptors, such as VCAM-1 and ICAM-1, respectively, and subsequent firm adhesion. Association between CD38 and CD49d has been shown to enhance binding of VLA-4 to VCAM-1. A distinctive macromolecular structure found in CLL cells is the invadosome, which is composed of several proteins, including VLA-4, CD44v, CD38, CXCR4, and MMP-9. This complex may have different functions as it is involved in different steps of CLL TEM and participates in the retention of CLL cells in the LNs.

In order to home into their protective niches, CLL cells hijack the physiological process of leukocyte TEM by taking advantage of the different adhesion molecules expressed on their surface (Figure 2). Not only do these adhesion molecules mediate the homing of malignant B cells into LN and BM, but they also provide essential survival signals, promoting CLL progression and resistance to therapy. In this section, we discuss the different classes of adhesion molecules that regulate these fundamental processes in CLL.

### 4.1. Selectins and Selectin Ligands

#### 4.1.1. L-Selectin

Early studies focusing on the correlation between the expression of different surface molecules and the migratory potential of CLL cells showed that low levels of L-selectin were linked to a defective TEM in CLL [121,122]. These observations are in line with a subsequent study showing that BCR engagement results in a downregulation of CXCR4 and L-selectin, which inhibits the egress of CLL cells from the LN, leading to an accumulation of leukemic cells in this protective niche [123]. Paradoxically, L-selectin seems to be overexpressed in CLL cells that reside in the LN and BM niches compared to those circulating in the blood stream, and the long-term culture of CLL cells in vitro is accompanied by an increase in L-selectin expression, suggesting a pro-survival role of this protein in the proliferation centers [124]. Recently, an in vivo functional study using intravital microscopy and multiphoton imaging has shown that L-selectin mediates the interaction between CLL cells and the high endothelial venules (HEV) of the LNs and is required for the first step of CLL TEM, indicating that L-selectin could represent a valuable target to inhibit CLL homing into the LNs [125].

#### 4.1.2. CD44

CD44 is a type I transmembrane glycoprotein whose principal ligand is the glycosaminoglycan hyaluronic acid (HA), although it can also interact with other extracellular matrix components, including osteopontin, fibronectin, laminin, and collagen [126,127]. A specific glycoform of CD44, known as HCELL, is also capable of binding to E- and L-selectins [128,129]. CD44 engagement by its ligands induces a vast array of intracellular pathways spanning from cell growth, survival, differentiation, and motility to the activation and homing of T lymphocytes as well as tumor development and metastasis [127,130,131,132]. Expression of CD44 is rather complex, as the *CD44* gene gives rise to many different variants by alternative splicing [133]. The CD44 isoforms include the standard version (CD44s) and larger variants, collectively referred to as CD44v, which are particularly frequent in tumor cells [134].

The different types of CD44 ligands, together with the presence of different splicing variants expressed by CLL cells [135], could explain the contradictory observations regarding CD44 expression and CLL prognosis [136]. To add another level of complexity, it has been shown that the CLL activation status influences the expression and the post-translational modifications of the CD44 variants, as CLL activation by CD40L induces the expression of CD44v3 and CD44v6 variants and also the N-linked glycosylation of the latter, which increases binding to HA and stromal cells [135]. In light of this evidence, it has been proposed that CD44 may function as a migration stop signal in CLL, impairing migration and inducing CLL cell arrest on HA-bearing cells in the microenvironment, thereby facilitating the interaction between CLL cells and CD4^+^ CD40L^+^ T cells in proliferation centers [137,138]. CD44 has also been implicated in the homing of CLL cells into the spleen, as genetic deletion or neutralization of CD44 with blocking antibodies impairs CLL accumulation in the spleen in vivo [138,139]. CD44 was also found to be present in a macromolecular complex together with CD38, VLA-4, and other proteins, which will be discussed in the CD49d and VLA-4 section.

Although the role of L-selectin and CD44 in CLL has been investigated mostly in the context of LN homing, there are no data regarding their possible involvement in BM homing as E-selectin ligands. L-selectin and CD44 can be decorated by sLe^a/x^, a modification that enables them to bind E-selectin, an important molecule serving as a BM homing receptor [140]. CLL cells express low levels of SLe^a/x^ [141,142]; despite the presence of different potential E-selectin ligand scaffolds, such as CD43, CD44, and L-selectin, binding to E-selectin should be severely compromised. It is conceivable that in CLL the selectin ligand functions may be carried out by integrins, which, while being the master mediators of firm adhesion, under some circumstances can also support tethering and rolling [143].

### 4.2. Integrins

#### 4.2.1. CD49d and VLA-4

CD49d, the α4 subunit of the VLA-4 integrin, is a strong independent predictor of overall and progression-free survival in CLL [144,145,146,147]. CD49d is expressed in approximately 40% of all CLL cases, with a validated cut-off of ≥30% [144,145]. While most cases (80%) express CD49d in a homogeneous pattern, few of them (20%) display a bimodal expression, with one population completely negative and the other one positive with a fluorescent signal well above the established cut-off [148]. Interestingly, the CD49d-positive population in the bimodal cases is characterized by an increased propensity to proliferate and a phenotype resembling that of LN resident cells [148], suggesting that CD49d may play an important role in the context of the LN and BM microenvironments and resistance to therapy. In line with this hypothesis is the observation that in patients with bimodal CD49d expression, Bruton’s tyrosine kinase inhibitors (BTKi), such as ibrutinib, induce an increase in the CD49d-positive population [148]. The importance of CD49d in CLL stems from the crucial role that VLA-4 plays in CLL extravasation. The homing of CLL cells in the BM and LN is largely dependent on VLA-4 [149,150,151]. Indeed, a higher expression of VLA-4 has been associated with an increased number of CLL cells in the BM during disease progression [152]. Thus, leukemic cells expressing CD49d are more likely to localize in lymphatic tissues, where they are protected from drug-induced cell death [148].

As with all integrins, VLA-4 shifts between low- and high-affinity conformations for ligand binding and, depending on the cell type, can be basally activated or inactivated [153]. The switch to an active VLA-4 conformation is induced by an inside-out signal emanating from different chemokines, including SDF-1α, which has been shown to be important in CLL homing and migration. Indeed, CXCR4, the receptor for SDF-1α, is expressed at high levels in CLL, and stimulation with SDF-1α induces the TEM of CLL cells in vitro [154]. Interestingly, CLL cells that have been induced by SDF-1α to transmigrate under a layer of stromal cells display high levels of CD49d, suggesting that CXCR4 and CD49d cooperate in this process [54]. Of note, there is a significant correlation between the CXCR4 and CD49d expression levels, further supporting the hypothesis that these molecules may be functionally linked [155]. CD49d also cooperates with other signaling molecules to induce TEM in CLL cells. It has been shown that CD49d is involved in CLL TEM mediated by CCL19 and CCL21, chemokines that are produced by HEV or by the surrounding LN stroma [156]. Moreover, CD49d and the autocrine production of VEGF by CLL cells are required for CCL21- and SDF-1α-mediated TEM through ECs [157]. Stimulation of VLA-4 by its natural ligands VCAM-1 and fibronectin enhances CLL secretion of the matrix metalloproteinase-9 proform (proMMP-9), a protease important in CLL TEM and invasion through the basement membranes [158,159]. Remarkably, proMMP-9 and its mature form localize to podosomes, which are induced by VLA-4 engagement, and promote degradation of the gelatin/fibronectin matrix [159]. In a subsequent study, it was shown that localization of proMMP-9 on the cell surface is mediated by VLA-4 and a high molecular weight CD44v, which form a molecular complex that binds to proMMP-9 [160]. All these data point out the relevant role of CD49d (and VLA-4) in different aspects of CLL TEM, including posodome formation and invasion.

CD49d has multiple levels of regulation in CLL, spanning from epigenetic mechanisms to protein–protein interactions and post-translational modifications. High levels of CD49d expression due to the hypomethylation of the *ITGA4* promoter, the gene encoding for CD49d, have been associated with trisomy 12 [161]. This association may explain the important LN adenopathy often observed in patients carrying the trisomy 12 abnormality and the tropism of these cells for the LN [162]. CD49d has been shown to physically interact with CD38 [163], another important prognostic marker in CLL. This interaction occurs in the context of a macromolecular complex that includes CD44, CXCR4, and MMP-9, a structure known as the CLL invadosome [164]. Importantly, the physical association between CD49d and CD38 enhances CLL adhesion to CD49d ligands such as VCAM-1 and fibronectin [163]. Finally, the function of CD49d is regulated by sialylation, a post-translational modification consisting of the attachment of terminal sialic acids to underlying glycans that decorate proteins and lipids. In CLL, removal of sialic acid by neuraminidase treatment leads to desialylation of CD49d and inhibition of VCAM-1- and fibronectin-dependent migration, adding a new layer of regulation to an already complex spectrum of VLA-4 modulations [142].

#### 4.2.2. β2 Integrins

The integrin subunit β2, also known as CD18, pairs with four different α subunits, generating the following integrins: LFA-1 (also known as CD11a/CD18, αLβ2), macrophage receptor 1 (Mac-1, also known as CD11b/CD18, αMβ2, complement receptor 3 [CR3]), complement receptor 4 (CR4, also known as CD11c/CD18, αXβ2, p150.95), and CD11d/CD18 (also known as αDβ2) [165]. β2-containing integrins are expressed exclusively on leukocytes and participate in cell trafficking, including adhesion, migration, and extravasation. A high expression of β2 integrins has been correlated with progressive disease [166]; however, in a cohort of 113 CLL patients, low expression levels of CD18 were correlated with advanced disease (Rai stage III–IV) and a diffuse BM pattern [167]. Indeed, compared to normal B cells, β2 integrins are barely detected in CLL [151]. Low expression levels of CD11a have been correlated with an impaired homing of PB-derived human CLL cells into murine LN and BM, which was exacerbated by the absence of CD49d, suggesting that these two integrins cooperate for an efficient homing of CLL cells into protective niches [151]. Interestingly, high expression levels of CD18 and CD11a have been found in CD38-positive CLL cells [151,168] and also in malignant cells harboring trisomy 12, similar to what has been observed for CD49d [169]. Moreover, it has been shown that the expression levels of CD18 are regulated by methylation of the promoter of the CD18-encoding gene *ITGB2*, a mechanism similar to the one modulating CD49d expression. However, low expression levels of CD18 may also be due to a specific variant found in CLL patients harboring a mutation that results in a single amino acid residue change from glutamate to lysine (E630K), which is predicted to alter the protein function [170]. Other β2 integrins that may contribute to CLL TEM are CR3 and CR4. Indeed, both integrins participate in CLL adhesion on fibrinogen [171,172], while only CR4 seems to be involved in SDF-1α-mediated migration on fibrinogen-coated transwell plates [171]. However, the contribution of these integrins to CLL TEM in vivo remains to be established.

#### 4.2.3. αVβ3 and α3β1

The integrin αVβ3 has been found to be expressed in CLL cells but not in normal B cells [173,174]. This integrin, also known as CD51/CD61, is the main receptor for vitronectin and participates in diverse biological processes such as angiogenesis, osteoclast-mediated bone resorption, and metastasis [175]. In CLL, integrin αVβ3 is mainly localized in the nucleus, with only small amounts present on the cell surface [173]. Both nuclear and membrane fractions have been found to be N-glycosylated and phosphorylated on tyrosine 759 in the cytoplasmatic tail of the β3 subunit. The latter modification seems to be indicative of an activated conformation [176], suggesting that this integrin may be constitutively active in CLL. Interestingly, immunoprecipitation studies have shown that in CLL, the nuclear fraction of integrin αVβ3 interacts with histone H3, suggesting a novel nuclear function for this integrin [173]. However, no functional studies were performed, and the role of the integrin αVβ3 in CLL biology remains to be determined.

Another integrin that is widely expressed in CLL is α3β1 [137]. Its expression, together with L-selectin and CD54, represents a good prognostic marker in CLL [177]. The presence of the α3β1 integrin enables CLL cells to migrate on laminin-332, particularly for cells with a mutated *IGHV* profile [108]. Since laminin-332 is present in the LN compartment, this protein may represent an additional factor that promotes CLL homing and retention in the LNs.

## 5. Cell Adhesion-Mediated Drug Resistance in CLL

Besides their crucial role in CLL homing and TEM, adhesion molecules are also involved in the microenvironment-mediated therapeutic resistance, either directly by evoking intracellular signaling pathways following binding to their specific ligands, which results in the expression of anti-apoptotic/pro-survival factors, or indirectly by simply lodging malignant cells in the microenvironment that provides all the necessary signals to sustain cell survival. Therefore, targeting the adhesion molecules in CLL could have multiple advantages in that it can block the homing and recirculation of CLL cells into protective niches, promote the egress of malignant cells from those niches into the PB, where they are more susceptible to chemotherapeutic agents, and finally inhibit microenvironment-mediated drug resistance.

**Figure 3:**
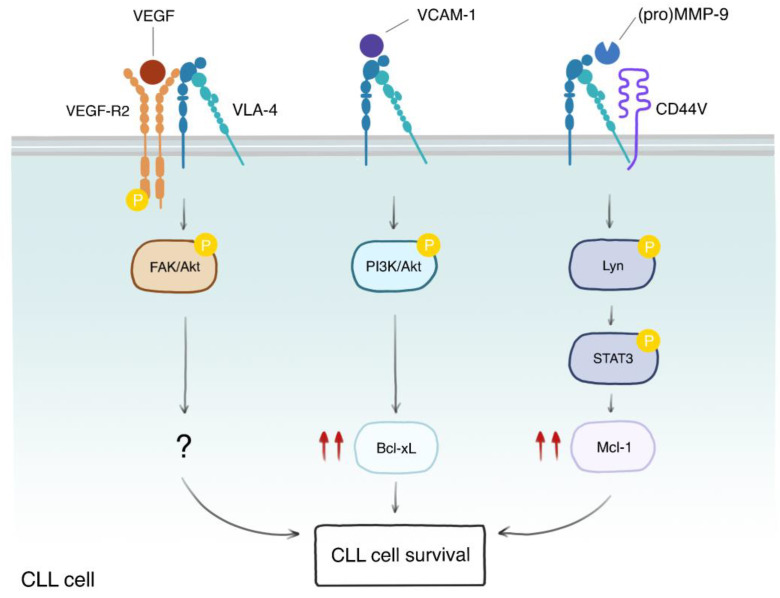
VLA-4 elicits different survival pathways depending on its binding partners and ligands. VLA-4 can be found in complex with different binding partners, such as VEGF-R2 and CD44v. Engagement of the VLA-4/VEGF-R2 complex by VEGF induces phosphorylation of VEGF-R2, which, in turn, promotes the phosphorylation and activation of FAK and Akt kinases, leading to CLL cell survival, though the downstream molecular players are not known (?). On the other hand, stimulation of VLA-4 by VCAM-1 activates the PI3K/Akt axes, leading to Bcl-xL upregulation (double red arrows) and survival. An additional survival pathway involves the induction of Mcl-1 through Lyn-mediated phosphorylation and activation of STAT3. This pathway is stimulated by the binding of either the pro-form or the mature form of MMP-9 to the VLA-4/CD44v complex. These data indicate that VLA-4 participates in multiple and different pathways that are important for microenvironment-mediated CLL survival.

As previously discussed, VLA-4 is the main integrin responsible for the interaction between CLL cells and the cellular as well as a-cellular components of the microenvironment, such as BM MSCs, FDCs, ECs, and fibronectin. VLA-4 is capable of reducing spontaneous apoptosis in vitro in CLL cells cultured in the presence of its recombinant ligands, such as VCAM-1, fibronectin, and the globular C1q (gC1q) domain of elastin microfibrilinterfacer-1 (EMILIN-1) [178,179]. Moreover, engagement of VLA-4 by recombinant fibronectin protects CLL cells from fludarabine-induced apoptosis, in particular cells expressing high levels of CD49d [155]. The mechanisms of fibronectin-induced protection from fludarabine involve the upregulation of Bcl-xL and downmodulation of TP53 [109,180]. VLA-4 cooperates with LFA-1 in mediating pro-survival signals from the BM MSCs and ECs that rescue CLL cells from spontaneous and drug-induced apoptosis [103,181]. Although these data indicate that VLA-4 and integrins protect CLL cells from apoptosis, one study reported that recombinant VCAM-1 and BM MSCs did not inhibit spontaneous apoptosis in both CD49d-positive and -negative isolated CLL cells [182], failing to demonstrate these effects. This discrepancy may be due to the specific time points chosen to evaluate apoptosis or to the different BM MSCs used to model the microenvironment. Besides its function in CLL TEM, binding of the proMMP-9 and its active form to the VLA-4/CD44 docking complex stimulates CLL survival [183]. This intracellular pathway relies on the upregulation of Mcl-1 induced by activation of the signal transducer and activator of transcription (STAT3) in a lymphocyte-specific protein tyrosine kinase (Lck)/Yes novel tyrosine kinase (Lyn)-dependent manner. This survival pathway is active in CLL cells regardless of their *IGVH* mutational status, CD38 and ZAP70 expression, and is independent of the MMP-9 catalytic activity. Of note, VLA-4 engagement by either MMP-9 or VCAM-1 has different outcomes, as MMP-9 promotes Mcl-1 upregulation while VCAM-1 induces Bcl-xL. [183]. Moreover, the binding of VCAM-1 to VLA-4 induces a different signaling pathway that relies on the activation of phosphoinositide 3′-kinase (PI3K)/Akt kinases. Interestingly, VLA-4 has been found to associate with VEGF receptor 2 (VGEF-R2), which seems to be necessary for the binding of CLL cells to VEGF [184]. Engagement of the VLA-4/VGEF-R2 complex by VEGF activates a survival pathway that involves the phosphorylation and activation of focal adhesion kinase (FAK) and Akt, though the downstream molecular players of this survival signaling cascade were not examined in this study [184]. All these data indicate that VLA-4 outside-in survival signals strongly depend on the type of ligands and binding partners (Figure 3). The association of CD38 and CD49d stimulates the adhesion of CLL cells to VCAM-1 and induces protection from spontaneous apoptosis. Indeed, CD49d/CD38 double-positive cells are more viable compared to CD49d-positive CD38-negative cells when cultured on recombinant VCAM-1, demonstrating a functional synergy between these two molecules. CD38 indirectly induces CD49d-positive CLL cell survival by increasing the expression of VCAM-1 on endothelial cells [77].

CD44 is another adhesion molecule capable of inducing survival signals in CLL cells. Microenvironmental stimuli that activate CLL cells, such as CD40L or contact with stromal cells, increase the expression of CD44 on CLL cells [135,139]. In the *Eμ-Tcl1* CLL mouse model, CD44 deficiency greatly impairs disease development by increasing apoptosis of leukemic cells due to a decrease in the expression levels of Mcl-1 [139]. In vitro, stimulation of CD44 by HA reduces spontaneous and fludarabine-induced apoptosis by increasing the levels of Mcl-1 [185]. CD44 is also responsible for FDC-mediated protection of CLL cells from spontaneous apoptosis, which is dependent on the induction of Mcl-1 [66].

## 6. Targeting the Cell Adhesion Molecules in CLL

**Figure 4:**
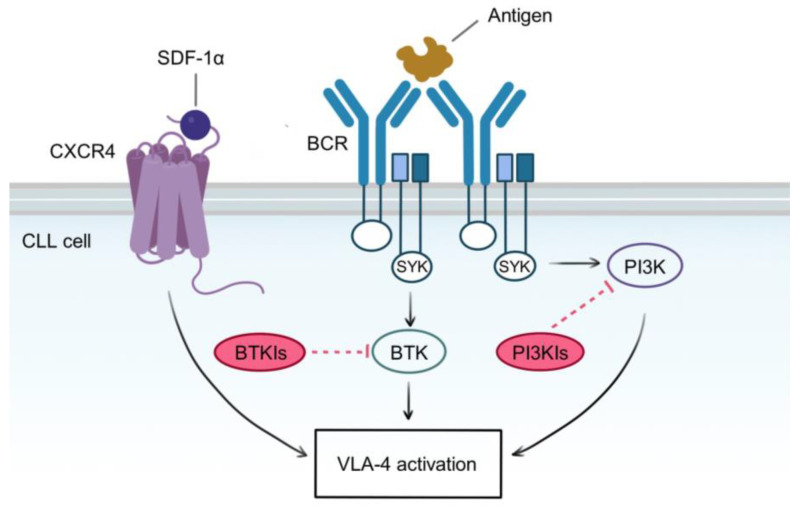
Molecular pathways involved in VLA-4 activation. Self or foreign antigens bind to BCR and induce its cross-linking and subsequent signaling pathways, leading to CLL proliferation, survival, and VLA-4 activation. These pathways are mediated by redundant kinases, whose activity is induced by a common BCR intracellular complex, containing the tyrosine kinase SYK. BTK and PI3K are probably the most well-described players in the BCR signaling cascade, and they are targeted independently by BTK and PI3K inhibitors. However, VLA-4 is also activated by an inside-out pathway emanating from CXCR4 stimulation, which is independent of BTK and PI3K. Since VLA-4 activation relies on all these redundant pathways, single or combined blockade of BTK and PI3K is insufficient to abrogate VLA-4 function. A therapeutic strategy aimed at completely inhibiting VLA-4 activity should target both BCR and CXCR4 axes.

Due to their role in TEM, adhesion, and CAMDR, it is not surprising that these adhesion molecules have been considered relevant targets for CLL treatment. Direct targeting of CD49d using the anti-VLA-4 antibody natalizumab has been explored only in vitro. In these studies, natalizumab was capable of overcoming stroma-mediated drug resistance to rituximab in a panel of B-lymphoma cell lines [186] and also decreased CLL migration in a physiologically relevant model of lymphocyte migration [187], demonstrating that direct targeting of CD49d may have therapeutic implications in CLL. However, modulation of adhesion molecules in CLL is mostly achieved indirectly by clinically relevant drugs, such as the BTKi ibrutinib. It has in fact been shown by our group that in patients treated with ibrutinib plus rituximab, there was a significant downmodulation of adhesion molecules, including CD38, CD43, CD44, CD62L, and CD49d [188]. In a follow-up study, it was also demonstrated that patients with a greater downmodulation of CD49d experienced a deeper and prolonged tumor burden reduction [189]. Indeed, ibrutinib and other BTKis, but also the spleen tyrosine kinase (SYK) inhibitor fostamatinib and the phosphoinositide 3′-kinase (PI3K) isoform p110δ (PI3Kδ) inhibitor idelalisib, have similar clinical manifestations consisting in a rapid resolution of lymphadenopathy and a redistribution of CLL cells from the tissues into the circulation accompanied by a transient lymphocytosis [190,191,192]. LN shrinkage and CLL re-localization can be explained by the disruption of CD49d-dependent adhesion induced by BTKi and idelalisib [190,193]. VLA-4-mediated adhesion is enhanced in CLL cells by antigen-dependent BCR engagement as well as CXCR4 stimulation through an inside-out signaling pathway that involves several kinases such as BTK, SYK, and PI3K [149,194,195,196]. This inside-out pathway provides the mechanistic link between the BTK inhibition and the observed lymphocytosis as BTKis block BCR-dependent BTK activation and the subsequent CD49d-mediated cell adhesion [194,197,198]. Notably, CD49d-positive CLL patients experience reduced LN shrinkage and lymphocytosis, and the degree of CD49d positivity correlates with an inferior response to BTKis, such as ibrutinib and acalabrutinib [194,195]. This inability of the BTKis to elicit a proper response in CD49d-positive patients was attributed to residual VLA-4 activity due to a BCR-independent signaling pathway, such as CXCR4-mediated VLA-4 activation, and a BCR/PI3K-dependent pathway that bypasses BTK. Indeed, the addition of PI3K inhibitors completely abolished BCR- but not CXCR4-dependent activation of VLA-4, suggesting that for proper VLA-4 inhibition, both BCR and CXCR4 should be targeted (Figure 4) [194,195].

It has been shown that ligation of the inactivated component protein 3 factor b (iC3b) to CR3 expressed on CLL cells inhibits spontaneous apoptosis in vitro in CLL cells, which was reverted by specific antibodies directed towards the αM chain of CR3, suggesting that iC3b could serve as an anti-apoptotic factor in CLL [199]. However, the clinical significance of this observation has yet to be determined. Another adhesion molecule that has been considered a candidate for therapeutic intervention in CLL is the selectin CD62L. It has been shown that CD62L expression increases during CLL culture in vitro and is highly expressed in LN in vivo [124]. Importantly, neutralizing CD62L using blocking antibodies promoted spontaneous as well as drug-induced apoptosis in vitro regardless of the presence of co-culture systems mimicking the microenvironment, particularly in CLL cells with an unmutated *IGVH* status. Another study showed that idelalisib induced a downregulation of CD62L in vivo in CLL patients, which is most likely associated with an increase in the rolling velocity and the subsequent impaired adhesion of CLL cells onto the HEV [125]. These data demonstrate the therapeutic potential of targeting CD62L, although its efficacy in the clinic requires further investigation.

## 7. Conclusions

CLL is a disease extremely dependent on the tumor microenvironment, a complex network of different cell types and ECM proteins that nurtures CLL cells with a myriad of pro-survival and proliferation signals, provided as membrane-bound and soluble molecules. All the different aspects of CLL biology, spanning from the homing into protective niches to CAMDR, are exquisitely orchestrated by a large number of cell adhesion molecules, which have been recognized to be central players in disease progression, chemoresistance, and relapse. At least one of these molecules, namely CD49d, has been extensively characterized functionally, and its clinical impact has been well established. For others, including L-selectin, CD18, and CD44, their functions are beginning to be uncovered, but their importance in the clinic, if any, is still lacking or contradictory. Moreover, some of the adhesion molecules have been used to monitor minimal residual disease, namely CD43, but their function remains elusive. Open questions also remain for CLL TEM in specific organs, including the BM. While the CLL TEM in LNs has been well characterized, the same process in the BM is still incompletely defined, in particular the role of the selectins. Finally, the study of adhesion molecules has also contributed to uncovering essential survival pathways in CLL, such as those triggered by the BCR and chemokines, allowing the development of novel kinase inhibitors. This new class of pharmacological compounds produces an impressive clinical response in part by modulating the levels or activity of the adhesion molecules. The clinical success of these kinase inhibitors may be due to their ability to target multiple pathways, leading to a global inhibition of different adhesion molecules at once. Whether this clinical response can be achieved by a more direct, targeted approach towards a specific adhesion molecule remains to be determined.

Adhesion molecules are master regulators of CLL TEM and CAMDR. Although our knowledge of their role in CLL biology has been steadily growing in the past few years, more work is required to deepen our understanding of the role of this fascinating class of molecules in CLL progression and relapse.
